# Molecular Targeting of VEGF with a Suramin Fragment–DOCA Conjugate by Mimicking the Action of Low Molecular Weight Heparins

**DOI:** 10.3390/biom11010046

**Published:** 2020-12-31

**Authors:** Jooho Park, Tae-Bong Kang, Ji-Hong Lim, Hyung-Sik Won

**Affiliations:** 1 Department of Biomedical Chemistry, College of Biomedical & Health Science, Konkuk University, Chungju 27478, Korea; jhlim@kku.ac.kr; 2 Department of Biotechnology, College of Biomedical & Health Science, Konkuk University, Chungju 27478, Korea; kangtbko@kku.ac.kr (T.-B.K.); wonhs@kku.ac.kr (H.-S.W.)

**Keywords:** VEGF, molecular targeting, drug development, suramin, heparin

## Abstract

Molecular targeting of growth factors has shown great therapeutic potential in pharmaceutical research due to their roles in pathological conditions. In the present study, we developed a novel suramin fragment and deoxycholic acid conjugate (SFD) that exhibited the potential to bind to the heparin-binding site (HBD) of vascular endothelial growth factor (VEGF) and to inhibit its pathogenic action for the first time. Notably, SFD was optimally designed for binding to the HBD of VEGF using the naphthalenetrisulfonate group, allowing to observe its excellent binding efficacy in a surface plasmon resonance (SPR) study, showing remarkable binding affinity (K_D_ = 3.8 nM) as a small molecule inhibitor. In the tubular formation assay, it was observed that SFD could bind to HBD and exhibit antiangiogenic efficacy by inhibiting VEGF, such as heparins. The cellular treatment of SFD resulted in VEGF-inhibitory effects in human umbilical vein endothelial cells (HUVECs). Therefore, we propose that SFD can be employed as a novel drug candidate to inhibit the pathophysiological action of VEGF in diseases. Consequently, SFD, which has a molecular structure optimized for binding to HBD, is put forward as a new chemical VEGF inhibitor.

## 1. Introduction

Uncontrolled blood vessel formation or angiogenesis is involved in pathological progression or tumor growth in the body [1,2,3,4]. A variety of antiangiogenic drugs, including antivascular endothelial growth factor (VEGF) antibodies, have been developed and approved by the U.S. Food and Drug Administration (FDA) for clinical use [5,6,7]. VEGF inhibitors, including bevacizumab (an anti-VEGF antibody), are widely used, with the global sales of VEGF-targeting drugs exceeding 10 billion dollars per year [8]. Additionally, as VEGF is known to interact with heparan sulfate, a component of the extracellular matrix (ECM), via its heparin-binding domain (HBD) that is positively charged with many arginine groups [9,10], the VEGF-binding effect of heparin or heparin sulfate has also received attention in many pharmaceutical studies to regulate or inhibit VEGF [11,12,13,14]. Accordingly, the anti-VEGF effect of heparin has been well-documented, thereby suggesting heparin as a promising drug candidate in VEGF-related therapy [15,16,17,18,19].

Heparin is a clinically used anticoagulant agent that is also prescribed to cancer patients to prevent blood coagulation [20]. Several kinds of heparins with different molecular sizes, heparin derivatives, and heparin conjugates have also been developed to inhibit VEGF in antiangiogenesis therapy; however, none of them has been used clinically [21]. Additionally, suramin, a small polysulfonated naphthylurea developed by Bayer, has gathered great attention due to its strong inhibitory effect on angiogenesis via VEGF inhibition, similar to the effect of heparin binding [22,23]. Several studies have demonstrated that suramin or suramin fragments (the key functional groups of suramin) can exhibit a strong anticancer effect by binding to HBDs in various growth factors [24,25]. Unfortunately, suramin is used only for the treatment of African sleeping sickness due to its severe toxic effect [26]. In addition, none of the known suramin analogs has been successfully developed into an anticancer medicine. Although a few synthetic suramin analogs possessing high antiangiogenic activity were previously chosen as drug candidates [27,28], they failed in clinical trials due to low efficacy and high toxicity [29].

In the case of heparin conjugates, bile acid-conjugated heparin molecules have been examined due to their effective antiangiogenic effect with low toxicity [30,31]. However, the strict approval process of the FDA has hindered the development of heparin conjugates due to the complexity of their macromolecule mixture [32]. As heparin is a mixture of large polysaccharides, accurate analysis and quality control in the manufacturing process of heparins are extremely difficult. In contrast, fondaparinux, a small-sized (1728 Da) synthetic pentasaccharide derived the high-affinity binding site in large heparin (avg. 12 kDa) marketed by GlaxoSmithKline, has been successful in the commercial development of heparin as an anticoagulant drug widely used in the clinical field [33]. However, in terms of anticancer therapy with FDA approval, no heparin-like anticancer agents are available due to their complicated synthesis and unwanted anticoagulant effects.

In this context, we previously evaluated various heparin and bile acid conjugates as VEGF inhibitors for anticancer therapy [30,34]. However, the chemical conjugation of heparin led to an increase in the molecular size of said heparin and its complexity. To overcome these limitations, we also developed smaller heparin-based conjugates, which were more favorable for VEGF inhibition [34]. As an alternative approach to developing more improved agents, in the present study, we generated a novel conjugate of small-sized synthetic suramin and deoxycholic acid (DOCA), reasoning that the suramin fragment (SF), similarly to heparins, could confer binding affinity to the HBD of VEGF, resulting in the inhibition of angiogenesis in tumors, and that DOCA conjugation to the SF would increase its binding affinity to VEGF. To prove this concept for suramin and DOCA conjugation, the molecular interaction of heparins and the suramin fragment–DOCA conjugate (SFD) with VEGF was investigated by surface plasmon resonance (SPR) and in silico simulation. We expect that this research will provide a molecular basis to overcome the fundamental problems in new drug development associated with the employment of functional macromolecules for VEGF inhibition.

## 2. Materials and Methods

### 2.1. Materials

The suramin fragment (disodium 8-amino-1,3,6-naphthalenetrisulfonate) was purchased from TCI (Tokyo, Japan). Deoxycholic acid (DOCA), dimethylformamide (DMF), formamide, unfractionated heparin, toluidine blue, and 1-ethyl-3-(3-(dimethylaminopropyl)carbodiimide (EDAC) were obtained from Sigma Aldrich (St. Louis, MO, USA). Low molecular weight heparin (Nadroparin) was obtained from the Nanjing King-Friend Biochemical Pharmaceutical Company Ltd. (Nanjing, China). Recombinant vascular endothelial growth factor 165 (VEGF) was purchased from Peprotech (Rocky Hill, NJ, USA). SPR sensor chips and running buffer were from GE Healthcare (Uppsala, Sweden). Endothelial growth medium-2 (EGM-2) was purchased from Lonza (Walkersville, MA, USA). Matrigel was obtained from BD Bioscience. All reagents were used without further purification.

### 2.2. Cell Culture

Human umbilical vein endothelial cells (HUVECs) were obtained from Promocell (Heidelberg, Germany). The cells were maintained and cultured in EGM-2 with SupplementMix (Promocell) that contained hydrocortisone, ascorbic acid, 2% fetal bovine serum (FBS) (GIBCO, NY, USA), and various growth factors, including human epidermal growth factor, vascular endothelial growth factor, and human fibroblast growth factor. The cells were incubated under a CO_2_ incubator with 5% CO_2_ (95% air) at 37 °C.

### 2.3. Synthesis and Characterization of SFD

Deoxycholic acid (100 mg) was prepared in DMF (0.9 mL) at room temperature. Heat-treated formamide (2 mL) was used to dissolve the hydrophilic suramin fragment (20 mg). Subsequently, the DMF and formamide solution were mixed together for 2 min. An excess amount (45 mg) of EDAC was added to the solution to initiate the amide formation reaction, followed by further incubation at room temperature for 12 h. Distilled water (10 mL) was added to the solution before filtration. The solution was freeze-dried to obtain powder, which was dissolved in distilled water. The mixture was precipitated in an acetone/ethanol (1:2) cosolvent to remove the unreacted materials. The reaction and purity of the synthesized suramin fragment and deoxycholic acid conjugate were monitored by thin-layer chromatography (TLC), followed by exposure to UV irradiation in a TLC-viewing UV cabinet. The molecular structure of the final product was confirmed by ^1^H NMR spectroscopy. ^1^H NMR spectra were recorded at 500 MHz using deuterium oxide (Sigma Aldrich) or dimethyl sulfoxide-d6 (Sigma Aldrich) as the solvent. The heparin fragments and heparin conjugates were prepared by the methods described previously [34,35].

### 2.4. In Silico Docking Calculations

The molecular structure of the HBD of VEGF (PDB code: 2VGH) was reported in a previous study [36]. The structure was visualized and optimized by the AutoDockTools package programs for the docking study. In the case of the molecular structure of the heparins, low molecular weight heparin (LMWH), LMWH–deoxycholic acid conjugate (LHD), heparin fragment–DOCA conjugate (HFD), and SFD were created using ChemDraw Professional 15.1 (Cambridge Soft, CA, USA). The molecular structures were converted and further stabilized by Chem3D 15.1 (Cambridge Soft, CA, USA). The molecular docking simulation between the biomolecules and VEGF was carried out with AutoDock Vina 1.0.3 [37,38]. In the docking setup, briefly, all rotatable bonds in the heparins or SFD remained free, while the HBD was kept rigid and the grid dimensions of 40 × 30 × 26 Å had the following coordinates: x = −4.3, y = 6.0, and z = 6.6. Finally, the volume of the binding pocket cavities of SFD was calculated using BIOVIA Discovery Studio 2020. The 3D molecular structure of the biomolecules or VEGF was simulated by BIOVIA Discovery Studio and the PyMOL program (PyMOL molecular graphics version 1.7.0.1; Schrödinger, LLC).

### 2.5. Surface Plasmon Resonance (SPR) Study

SPR binding analysis methodology was used to study the molecular interactions between VEGF and the biomolecules. The binding affinity was measured using a Biacore T100 instrument (Biacore AB, Uppsala, Sweden). Briefly, the recombinant human VEGF_165_ (Peprotech, Rocky Hill, NJ, USA) was immobilized on the gold surface of a CM5 chip (Biacore carboxymethylated dextran sensor) by a standard EDC/NHS coupling reaction [39]. Affinity analysis of SPR was performed for the heparins, HFD, and SFD at concentrations ranging from 0.001 to 1 μM (LMWH, 4500 Da; LHD, 6000 Da; HFD, 2500 Da; SFD, 802 Da) in HEPES-EP running buffer (HEPES 10 mM, sodium chloride 150 mM, EDTA 3 mM, and 0.05% P-20) [34]. Binding analysis was carried out at a flow rate of 20 μL/min (37 °C), and VEGF protein was regenerated with an NaOH solution (50 mM) for 5 s. The binding data were analyzed for affinity characteristics using the Biacore T100 evaluation software. The curves in the figure were fitted according to the 1:1 Langmuir binding model using the Biacore software.

### 2.6. Cell Viability Test (CCK Assay)

To assess the cytotoxicity of SFD and the other molecules, HUVECs were cultured on a 96-well plate. When the 96-well plate was confluent with the HUVECs, the media were replaced with endothelial growth medium-2 (EGM-2) without growth factors containing different concentrations (200 and 800 μg/mL) of LMWH, LHD, HFD, and SFD. After incubation at 37 °C for 12 h, the EGM MV2 media were removed, and EBM (endothelial basal medium) media with 10 μL of cell counting kit-8 (CCK-8) solution was added to the plate to analyze the viability for 1 h. The HUVEC viability was calculated from the absorbance value (450 nm) using an ELISA reader (*n* = 6).

### 2.7. Endothelial Tubular Formation Assay

Initially, the HUVECs were cultured in T-flask with supplemented EGM MV2 media for 4 days. Then, the cells were detached by EDTA/trypsin treatment and placed on a Matrigel-coated (for 30 min at 37 °C) 96-well plate (2 × 10^4^ cells per well). The cells were cultured in 100 μL of EGM MV2 media containing VEGF_165_ (60 ng/mL) and 5% FBS with LMWH, LHD, HFD, or SFD (50 μg/mL). After 6 h of incubation at 37 °C, Calcein AM (Sigma Aldrich) was added for 30 min to visualize the endothelial tubular formation of the HUVECs. The number of completed vessels in the field was counted via confocal laser scanning microscopy (CLSM) (*n* = 4).

### 2.8. Wound Healing Assay

The HUVECs were seeded in a 24-well plate after EDTA/trypsin treatment. The cells were incubated in supplemented EGM MV2 media until the cells reached confluence. The wound of the HUVECs was made uniform by a 1 mL tip at the center of each well. After washing 3 times with an EBM solution to eliminate cell debris, the remaining cells were incubated in EGM MV2 media (40 ng/mL VEGF and 5% FBS) for 24 h in the presence of different concentrations (40 or 400 μg/mL) of SFD. Subsequently, the supernatant was removed and the cells were fixed with a cold 4% paraformaldehyde solution for 10 min. After washing 3 times, the migrated cells were visualized by treatment with 0.001% toluidine blue (Sigma Aldrich), and the wound healing area was measured using ImageJ (U.S. National Institutes of Health) (*n* = 3).

### 2.9. Statistical Analyses

All statistical analyses were performed using SigmaPlot 13 Statistics (Systat Software Inc., San Jose, CA, USA). The difference between groups was measured by one-way analysis of variance followed by Bonferroni tests.

## 3. Results

### 3.1. Design and Characterization of SFD

**Figure 1:**
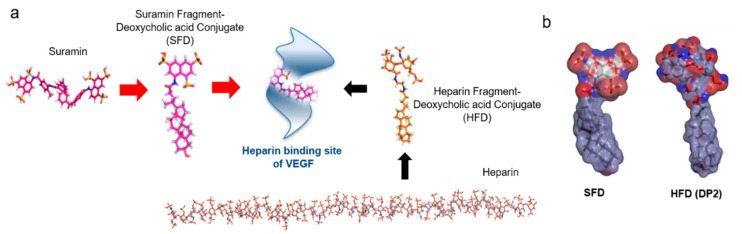
(**a**) Schematic rational design of a suramin fragment and deoxycholic acid conjugate (SFD) representing binding to the heparin-binding site of vascular epithelial growth factor (VEGF) compared to a heparin fragment deoxycholic acid conjugate (HFD). (**b**) The surface charge of the SFD and HFD show similarities in molecular binding.

Previously, it was reported that a series of heparin–DOCA conjugates could be employed as potent inhibitors of VEGF activity. In the present study, we developed a small synthetic anticancer agent to block the angiogenesis process in tumors. As heparins can bind to the heparin-binding site of VEGF under physiological conditions, we designed a novel small heparin-like angiogenesis inhibitor using a suramin fragment, as shown in Figure 1. To improve the existing knowledge on the therapeutic activity of heparin–DOCA conjugates, we developed a new small conjugate that can bind to the heparin-binding site of VEGF, thereby eliminating the complexity of heparins. The molecular length of SFD is approximately 19 Å, and since the length of HBD (15–25 Å) in VEGF is similar in size, it is considered optimal for VEGF binding. The synthesis of SFD using a suramin fragment and DOCA can be completed simply in one step and does not involve the complexity of macromolecules, unlike heparins (Figure S1). After synthesis and purification, the DOCA moiety in the SFD structure was confirmed by 1D proton NMR analysis (Figure S2). The molecular structure and surface charge of two biomolecules showed that the suramin fragment in SFD enabled bonding with the heparin-binding site of VEGF as a heparin mimic; hydrophobic DOCA served to strengthen the bonding between them (Figure 1b).

### 3.2. Docking Analysis with the Heparin-Binding Site of VEGF

**Figure 2:**
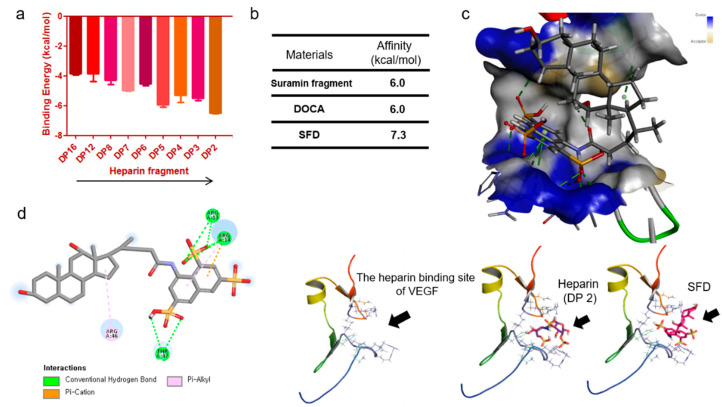
A binding simulation study with the molecular structure of the heparin-binding site in VEGF. (**a**) Calculated binding affinity for the selected heparin polysaccharides with different degrees of polymerization (DPs) ranging from 2 to 16. Smaller (low DP) heparins tend to show lower binding energy. (**b**) The enhanced binding energy of SFD to the heparin-binding site of VEGF was particularly evident in the simulation results. SFD is suitable for coupling to VEGF as it has both a suramin fragment (heparin mimic) and deoxycholic acid (DOCA). (**c**) Binding simulation for a heparin fragment (DP 2) or SFD with VEGF represents that SFD has a proper molecular size and structure suitable for binding. In particular, the suramin fragment in SFD exhibits a number of hydrogen bonds with HBD, while DOCA shows a strong hydrophobic interaction. (**d**) The energy-minimized conformation of SFD in the binding site of HBD. There are various pi–pi interactions between SFD and VEGF. In particular, hydrophilic peptides including arginine in HBD exhibit various kinds of interactions with SFD. The molecular binding interactions of SFD were calculated using BIOVIA Discovery Studio (2020).

To determine the proper size of heparins for binding to VEGF, we studied the molecular basis of the interactions between heparin and the heparin-binding site of VEGF. We screened the binding energy of differently sized heparins by molecular docking simulation. The docking of various small-sized heparins was tested, and as a result, small heparin molecules were found to exhibit relatively stronger binding to the heparin-binding site of VEGF (Figure 2a). LMWH, the most commonly used heparin, has an approximate degree of polymerization (DP) of 16 but is larger for binding to the heparin-binding site of VEGF. Molecular binding studies suggest that small heparins (DP 2–5) have a higher binding affinity for VEGF (from −5.6 to −6.8 kcal/mol) than LMWH (−3.8 kcal/mol). As the length of the heparin oligosaccharide decreased from DP 16 to DP 2, the binding energy decreased, indicating that they can be optimized (to be smaller in size) to bind to the heparin-binding site. Moreover, the suramin fragment and DOCA showed a low binding affinity (−6.0 kcal/mol) due to their heparin-like property and strong hydrophobic interaction, respectively (Figure 2b). In particular, SFD showed relatively strong binding (low binding energy value) to VEGF, as it was able to bind to the heparin-binding site of VEGF via the suramin fragment and hydrophobic DOCA, stabilizing their binding. As shown in Figure 2c, SFD is of the appropriate size to bind to the heparin-binding pocket in the VEGF molecular structure; the binding of SFD with VEGF displayed multiple intermolecular interactions. The docking result suggests that the 8-amino-1,3,6-naphthalenetrisulfonate group (suramin fragment) can occupy the pocket of HBD while keeping hydrophobic interactions of DOCA without steric conflicts. Most of the interactions were conventional hydrophilic hydrogen bonds, although residues Arg46 and Arg14 showed pi-alkyl interactions (Figure 2d). In particular, the Arg31 and Arg14 of HBD show intermolecular hydrogen bonds with SFD. Taken together, the simulation results indicate that the small synthetic SFD can be employed as a structurally designed drug candidate for the inhibition of VEGF to regulate angiogenesis.

### 3.3. Surface Plasmon Resonance (SPR) Binding Study with VEGF

**Figure 3:**
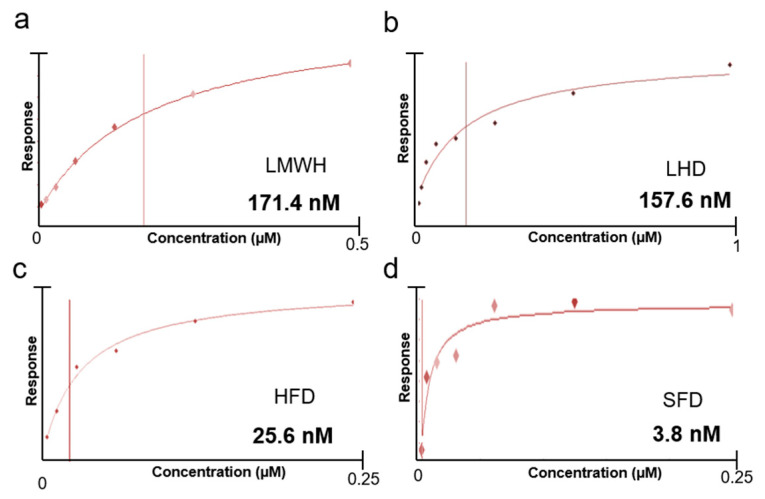
Relative surface plasmon resonance (SPR) responses for (**a**) low molecular weight heparin (LMWH), (**b**) low molecular weight heparin and DOCA conjugate (LHD), (**c**) heparin fragment and DOCA conjugate (HFD), and (**d**) SFD in HEPES (hydroxyethyl piperazineethanesulfonic acid)-buffered solution immobilized with VEGF on a CM5 chip. The affinity curves were plotted by using the Biacore T100 evaluation software.

In order to determine the specificity of SFD to VEGF, we evaluated the binding affinity of the heparins and SFD by surface plasmon resonance analysis. The dominant molecular interaction of various heparins with VEGF has been confirmed previously in many structure analysis studies on VEGF [40]. In this study, we designed a new heparin-mimic DOCA conjugate (SFD) and heparin fragments (HFs) or a heparin fragment and DOCA conjugate (HFD) were compared with SFD. Initially, VEGF was bound on the CM5 gold SPR chip, and then heparin, HFs, HFD, or SFD were allowed to flow to calculate the actual binding energy with VEGF. Consequently, the K_D_ (dissociation constant) value of the low molecular weight heparins (171.4 nM) and the heparin fragments (157.6 nM) showed high affinities for VEGF_165_, indicating that the heparins were able to bind to the heparin-binding domain of VEGF (Figure 3a,b). HFD had a higher binding affinity for VEGF (K_D_ = 25.6 nM) than the low molecular weight heparin or heparin fragments due to the presence of the DOCA moiety. Bile acid conjugation usually improves the binding affinity or inhibitory effect of heparins to VEGF (Figure 3c) [41,42,43]. In the case of SFD, it showed the lowest K_D_ value (3.8 nM) against VEGF, indicating that it could be an effective VEGF inhibitor for antiangiogenic effects (Figure 3d). SFD has three sulfone groups, similarly to heparin fragments, which can stably attach to the heparin-binding site and exhibit steady binding to VEGF.

### 3.4. Evaluation of the Antiangiogenic Effect of SFD on HUVECs

**Figure 4:**
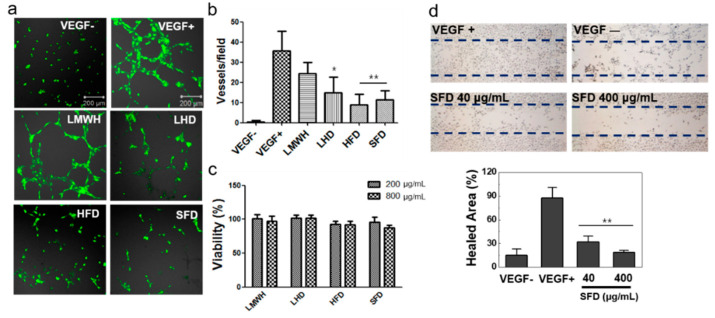
Inhibition of angiogenesis by heparins and SFD in the human umbilical vein endothelial cell (HUVEC) assay. (**a**) In vitro tubular formation of the HUVECs is presented after Calcein-AM treatment. (**b**) The number of vessels on the HUVECs was measured in the presence of LMWH, LHD, HFD, or SFD (*n* = 4). (**c**) The cytotoxicity test (CCK assay) demonstrated that the heparin conjugates and SFD had no cytotoxic effect on the cells (*n* = 6). (**d**) The VEGF-mediated wound healing assay showed that SFD can inhibit endothelial cell migration into the wound. The relative healed wound area was measured after toluidine blue treatment by using the ImageJ program (U.S. National Institutes of Health) (*n* = 3). * *p* < 0.05 vs. control group; ** *p* < 0.01 vs. control group.

The HUVECs were used to investigate the antiangiogenic effect of SFD in vitro. Initially, the HUVECs were incubated with or without VEGF on Matrigel to determine the mechanism related to the angiogenic properties of VEGF. As expected, VEGF clearly promoted the angiogenic process and increased blood vessel formation in vitro (Figure 4a). To confirm the inhibitory effect of heparin, LHD, HFD, and SFD, they were treated with VEGF to HUVECs. As a result, the relative vessel formation in the HUVECs was decreased by 68.5% (LMWH), 41.9% (LHD), 25.2% (HFD), and 32.2% (SFD) in comparison to the VEGF-treated group (100%) (Figure 4b). When VEGF was not treated, scarce vessel formation by the HUVECs was noted (1.4%). Moreover, the cytotoxicity of heparin, LHD, HFD, and SFD was tested with the HUVECs by CCK assay (cell counting kit-8 assay; viability test). No cytotoxicity at low (200 μg/mL) and high (800 μg/mL) concentrations was observed, indicating that they inhibited the angiogenesis process with low toxicity (Figure 4c). Finally, in order to study the manner in which SFD affected VEGF-mediated endothelial cell migration, we evaluated the antiangiogenic effect of SFD on VEGF-mediated wound healing assay. In the wound healing process induced by VEGF, SFD showed inhibitory effects at low (63.5% at 40 μg/mL) and high (78.4% at 400 μg/mL) concentrations, proving its notable antiangiogenic effect (Figure 4d).

## 4. Discussion

Initially, we carefully studied the molecular structure of VEGF to develop an efficient antiangiogenic agent that could inhibit its action. Subsequently, we obtained the clear structure of the heparin-binding site of VEGF, and it was obvious that its heparin-binding site was small compared to the molecular size of unfractionated heparins [34,36]. Therefore, small heparins or heparin fragment conjugates, such as low molecular weight heparin–DOCA (LHD) or heparin fragment–DOCA (HFD) conjugates, showed effective therapeutic effects. Despite the efficacy of heparin in biomedical science, its pharmaceutical use has faced challenges due to lack of approval of from the FDA. Heparin-based VEGF-specific drug development needs to be utilized from early drug discovery to development. To develop a drug that effectively binds and inhibits the heparin-binding site of VEGF, we redesigned the conjugate using a suramin fragment, as shown in Figure 1, because suramin is one of the most significant heparin mimics showing remarkable antiangiogenic effects in various studies [44].

The newly developed SFD is an optimized synthetic material for binding to the heparin-binding site of VEGF. Because heparin itself cannot be developed as a VEGF-targeting drug due to its complexity and anticoagulant properties, we propose that SFD can be employed as an attractive VEGF-inhibitory agent based on its potential to overcome the current problems of heparin conjugates. In this study, our data from the computer simulation and SPR binding studies with heparins and SFD clearly indicate that SFD is an effective biomolecule against the heparin-binding site of VEGF. The binding affinity results offer reasonable insight into the binding properties of SFD with VEGF under physiological conditions. We also compared heparin compounds with SFD by evaluating the antiangiogenic effect of biomolecules in HUVECs.

In this study, we evaluated the VEGF-inhibitory effects of the heparin mimic properties of SFD on VEGF-mediated blood vessel formation in primary HUVECs. Initially, treatment with high concentrations (200 and 800 μM) of SFD or heparins had no severe cytotoxic effect on the HUVECs. However, SFD suppressed VEGF-induced vessel formation in the HUVECs similarly to other functional heparin derivatives and conjugates. It was obvious that SFD inhibited the angiogenesis process of endothelial cells and affected vascular formation in the tumor model by inhibiting VEGF. Unlike other heparins, SFD has a clear molecular structure, which improves its binding ability to VEGF, thereby showing is efficiency as a potential drug candidate to inhibit VEGF in various diseases.

## 5. Conclusions

It is proposed that the newly developed VEGF inhibitor, SFD, synthesized by the conjugation of a suramin fragment with DOCA to achieve enhanced binding affinity, could be a potential drug candidate. Its VEGF-inhibitory property indicates that it could be applied as a novel therapeutic biomolecule in clinical settings by inhibiting the VEGF activity in patients. Above all, owing to the high anticoagulant effect of heparin and heparin conjugates, we expect that SFD can be successfully used in the treatment of VEGF-related diseases such as anticancer therapy. The presented rational drug design strategy is believed to offer great potential in the development of therapeutic VEGF-inhibitory agents with promising clinical applications.

## Data Availability

Data is contained within the article.

## Supplementary Materials

The following are available online at https://www.mdpi.com/2218-273X/11/1/46/s1, Figure S1: Synthetic scheme of suramin fragment and deoxycholic acid conjugate (SFD). Figure S2: 1H-NMR spectra of SFD, suramin fragment and DOCA.
