# Supplementary material for: Molecular Targeting of VEGF with a Suramin Fragment–DOCA Conjugate by Mimicking the Action of Low Molecular Weight Heparins
Source: Biomolecules. 2020 Dec 31;11(1):46. doi: 10.3390/biom11010046 (PMC7823656; doi:10.3390/biom11010046)
Supplement: Supplementary file 1 [file biomolecules-11-00046-s001.pdf]

Supplementary Information

Molecular Targeting of VEGF with a Suramin Fragment–DOCA Conjugate by Mimicking the Action of Low Molecular Weight Heparins

Jooho Park[1]*, Tae-Bong Kang[2], Ji-Hong Lim[1] and Hyung-Sik Won[2]

[1]Department of Biomedical Chemistry, College of Biomedical & Health Science, Konkuk University, Chungju 27478, Chungbuk, Republic of Korea; pkjhdn@kku.ac.kr (J. P)
[2]Department of Biotechnology, College of Biomedical & Health Science, Konkuk University, Chungju 27478, Chungbuk, Republic of Korea; kangtbko@kku.ac.kr (T.B.K.)
*Correspondence: pkjhdn@kku.ac.kr; Tel.: +82-43-840-3574 (J.P.)

S1. Results and Discussion

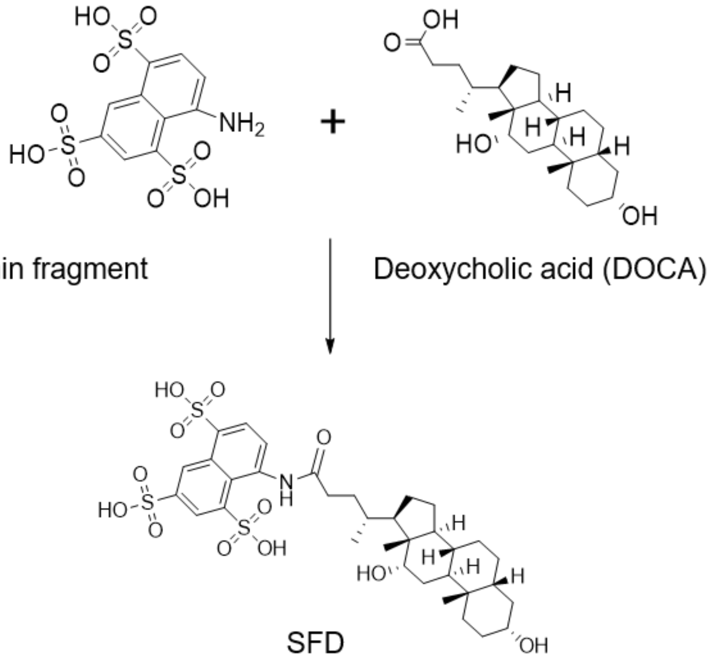

Figure S1. Synthetic scheme of suramin fragment and deoxycholic acid conjugate (SFD). Suramin fragment was conjugated to deoxycholic acid (DOCA) in one step.

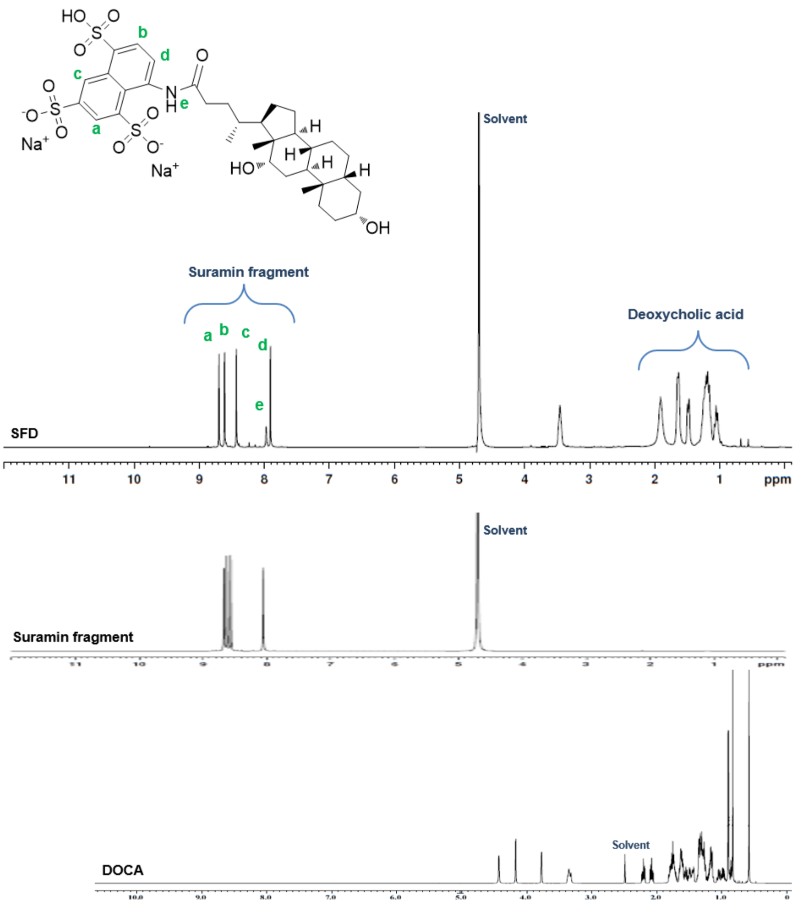

Figure S2. ^{1}H-NMR spectra of SFD (deuterium oxide as solvent), suramin fragment (deuterium oxide as solvent) and DOCA (dimethyl sulfoxide-d6 as solvent) (500 MHz).
